# Case Report: Early-onset mevalonic aciduria in neonates with inflammatory marker elevated

**DOI:** 10.3389/fimmu.2026.1773017

**Published:** 2026-03-05

**Authors:** Shanshan Xue, Wujuan Shi, Xiaobo He, Lihong Hao

**Affiliations:** 1 Department of Neonatology, Tianjin Children’s Hospital, Tianjin, China; 2 Department of Neonatology, Children’s Hospital, Tianjin University, Tianjin, China

**Keywords:** inflammatory markers, mevalonate kinase, mevalonic aciduria, neonate, whole-genome sequencing

## Abstract

**Purpose:**

The aim of this study was to present a case of early-onset mevalonic aciduria (MA) in a neonate and summarize the relevant phenotypic and genotypic spectra of MA.

**Methods and results:**

We describe a neonate who presented with elevated inflammatory marker after birth. Liver function tests revealed liver injury, and enzyme-linked immunosorbent assay (ELISA) confirmed increased mevalonic acid levels in blood and urine. Whole-genome sequencing identified a novel homozygous mutation (c.928G>A, p.Val310Met) in the *MVK* gene. To date, only 16 neonate cases of MA have been reported in the literature. Affected individuals typically present recurrent fever, hepatosplenomegaly, lymphadenopathy, vomiting, diarrhea, and neurological damage symptoms. This case emphasizes that in patients presenting with recurrent fever accompanied by vomiting, diarrhea, hepatosplenomegaly, and lymphadenopathy, clinicians should pay close attention to differentiating MA from infectious diseases and autoinflammatory disorders to avoid misdiagnosis or underdiagnosis.

**Conclusion:**

We report one of the youngest neonates with early-onset MA diagnosed promptly, caused by the novel homozygous *MVK* variant, c.928G>A (p.Val310Met), and expand the genotypic and clinical phenotypic spectrum of *MVK* variants related with MA.

## Introduction

Infection is the most common cause of elevated inflammatory markers and/or fever in neonates. Meanwhile, a systemic inflammatory response may also result from various non-infectious conditions, such as perinatal asphyxia, meconium aspiration syndrome (MAS), or surgery. The clinical manifestations of inflammation can usually be resolved with the early initiation of appropriate antibiotic therapy. When a neonate presents with chronic or recurrent systemic inflammation, autoinflammatory diseases (AIDs) should be considered because of their potentially devastating consequences. Mevalonic aciduria (MA) is one of the classical monogenic hereditary AIDs characterized by recurrent fever, hepatosplenomegaly, lymphadenopathy, vomiting, diarrhea, and neurological impairment, which is presumably associated with mevalonate kinase (*MVK*) gene mutations (1). These phenotypes belong to heterogeneous clinical manifestations of AIDs, resulting from pathogenic variations in genes of the innate immune system. The limited characterization of neonatal-onset MA coupled with its frequent clinical overlap with neonatal infections makes it difficult to avoid misdiagnosis or underdiagnosis. Herein, we report a case of MA presenting at birth with mild symptoms and signs suggestive for perinatal infection. This report aims to further expand existing knowledge by presenting clinical manifestations of early-onset MA in neonates and integrating the relevant phenotypic and genotypic spectra of MA.

## Case presentation

The patient was a male neonate born at 38^4/7^ weeks’ gestation via vaginal delivery at an external hospital. He was admitted to the neonatal intensive care unit (NICU) due to mild asphyxia at birth, with Apgar scores of 7 (1 min) - 7 (5 min) - 9 (10 min). Gentle oropharyngeal and nasal meconium suctioning was performed using a bulb syringe, and resuscitation was successful with subsequent positive pressure ventilation. Unfortunately, cord blood gas results were not available. On postnatal day 1, the neonate developed a fever of 37.8°C, accompanied by an elevated C-reactive protein (CRP) level (29.39 mg/L) and thrombocytopenia (78 × 10^9^/L). These findings were initially attributed to neonatal sepsis secondary to MAS, and antibiotic therapy was therefore initiated. On day 4 of antibiotic administration, the neonate achieved afebrile status and the CRP level decreased to 15.2 mg/L, indicating a partial response to the treatment. Antibiotic therapy was subsequently discontinued after a 7-day course. However, the next day following antibiotic withdrawal, the neonate developed recurrent fever, with the CRP level rebounding to 18.6 mg/L. Given this clinical recurrence, nosocomial infection was clinically suspected, and the neonate was transferred to our hospital.

Upon admission to our hospital, alternative antibiotic regimens were reinitiated. On physical examination, morbilliform rash and petechiae were observed throughout the face and body, along with hepatosplenomegaly (5 cm below the costal margin) and painless lymphadenopathy of the cervical, axillary, and inguinal regions. Hepatosplenomegaly was confirmed by both physical examination and abdominal ultrasound, with the liver and spleen each extending 5 cm below the costal margin. Clinical evaluation, ultrasonography, and standard cranial magnetic resonance imaging (MRI) were performed. Written informed consent for diagnostic genetic testing and publication of clinical information was obtained from the patients’ parents in accordance with the Declaration of Helsinki. Routine blood tests revealed fluctuations in the following parameters: serum CRP (11.35–47.1 mg/L), white blood cell count (11.36–21.39 × 10^9^/L), hemoglobin (62.1–195 g/L), and platelet count (52–189 × 10^9^/L) (**Supplementary Figures 1A–C**); however, serum procalcitonin levels remained within the normal reference range. Additional laboratory findings were as follows: alanine aminotransferase 173 U/L, aspartate aminotransferase 126 U/L, cholesterol 3.11 mmol/L, triglycerides 2.5 mmol/L, PCT 0.27 ng/mL, erythrocyte sedimentation rate 19 mm/h, and ferritin 765.6 ng/mL. Tests for antinuclear and extractable nuclear antigen antibodies were negative. Cytomegalovirus (CMV) DNA was detected at 1.1 × 10³ copies/mL, whereas the CMV pp65 antigen test result was negative. No other pathogens were identified. Ultrasonography revealed hepatosplenomegaly and lymphadenopathy. Head MRI suggested subdural effusion, left ventricular dilatation, and extracerebral space widening. Lung CT revealed scattered inflammatory consolidation in both lungs. Brainstem auditory evoked potentials revealed abnormalities in the right peripheral segment, consistent with mild-to-moderate, right-sided hearing loss. No abnormalities were detected on the ophthalmological examination.

Despite 2 weeks of treatment with various antibiotics (e.g., cephalosporins, meropenem, and linezolid), the therapeutic response remained unsatisfactory. The neonate persisted with recurrent fever, feeding difficulties, intermittent diarrhea, hepatosplenomegaly, and sustained elevation of inflammatory markers. The patient received twice red blood cell transfusions for anemia management and two doses of intravenous immunoglobulin (IVIG) for presumed refractory infection, whereas no platelet transfusion was administered. After 2 weeks of ineffective antibiotic therapy, an extensive diagnostic workup was initiated. The levels of urinary mevalonic acid were measured by gas chromatography–mass spectrometry (GC-MS; Agilent Technologies Inc. 5975C/7890A, USA) (2), which indicated that the mevalonic acid level in the patient’s urine was 4,750 mM/M creatinine (normal value <0.1 mM/M creatinine). In accordance with the clinical diagnostic criteria for MVK deficiency (MKD) formulated by the European Alliance of Associations for Rheumatology in 2015, the neonate’s clinical manifestations were scored, resulting in a total of 49 points. The scoring breakdown was as follows: age at onset < 2 years (10 points), generalized enlargement of lymph nodes or splenomegaly (8 points), intermittent diarrhea (20 points), and absence of chest pain (11 points) (3). The clinical diagnosis of MKD was suspected.

Previous studies have reported that mildly increased mevalonic acid excretion is observed in patients with the hyperimmunoglobulin D syndrome phenotype during febrile episodes, whereas severely affected patients with MA exhibit relatively low mevalonic acid excretion (4, 5). Therefore, the concurrent initiation of enzyme-linked immunosorbent assay (ELISA) and whole-genome sequencing (WGS) were performed for the confirmation of final diagnosis. ELISA results were available 2 days prior to genetic testing. Serum and urinary mevalonic acid levels were measured with the Human MVA ELISA Kit (Wuhan Mosak Biotechnology Co., Ltd., Wuhan, China). ELISA results demonstrated significantly elevated levels of mevalonic acid and mevalonolactone in both blood and urine, while MVK enzyme activity was reduced and immunoglobulin D (IgD) level was normal. The mevalonic acid levels in blood and urine were 425 and 390 ng/mL, respectively, which were both significantly higher than those of his parents (normal value <10 ng/mL). Peripheral blood samples were collected from the neonate and his parents, and WGS was performed using a high-throughput sequencing platform (MyGenostics, Beijing, China). Briefly, genomic DNA was fragmented, and sequencing libraries were constructed. Subsequently, DNA from the exonic regions and adjacent splice regions of target genes was captured and enriched with the GenCap custom enrichment kit (MyGenostics, Beijing, China) in accordance with the manufacturer’s protocol. Finally, the enriched libraries were sequenced on an Illumina NovaSeq 6000 sequencer (Illumina, San Diego, CA, USA) for paired-end reads of 150 bp. The quality control metrics of the sequencing data were as follows: the average sequencing depth of the target region was 49.11 Mb, and the proportion of loci with an average depth of >100× in the target region was 96.53% (10×) and 92.91% (20×). WGS and Sanger sequencing identified a homozygous mutation (c.928G>A, p.Val310Met) in the *MVK* gene, and his parents were found to carry a heterozygous variation at this locus (**Supplementary Figures 2 A–C**). According to the interpretation guidelines of the American College of Medical Genetics and Genomics, this variant was classified as pathogenic (PM1 + PM2 + PM3_Strong + PM5 + PP3).

After confirmation of the final diagnosis, the patient received adequate nutritional support with a deeply hydrolyzed protein formula, combined with symptomatic and supportive care. These included hepatoprotective therapy with glucurolactone and bicyclol to improve liver function, smectite for intestinal mucosal protection, probiotics for intestinal flora regulation, and vitamin K_1_ supplementation, all aimed at maintaining stable internal homeostasis. Additionally, glucocorticoid therapy with prednisone acetate (0.91 mg/kg/day) was initiated and continued for 7 days. Following this intervention, the patient’s body temperature normalized, liver function improved, and rash dissipated. However, inflammatory markers demonstrated recurrent elevation, and the patient exhibited poor weight gain. During the 67-day period of intermittent antibiotic therapy, the neonate presented with symptoms of hereditary recurrent fever, poor feeding, intermittent diarrhea, and drowsiness. The neonate was hospitalized and designated as patient 11 (**Table 1**).

**Table 1:** Summary of the genotypic and clinical phenotypic data in neonates.

Patient	P1	P2	P3	P4	P5	P6	P7	P8	P9	P10	P11	P12	P13	P14	P15	P16	P17
Origin	Germany	Germany	China	Italy	New Zealand	Germany	Morocco	Germany	Moroccan	America	China	Spain	Italy	America	Germany	France	
Sex	F	M	M	M	M	M	F	M	F	F	M	M	M	F	M	F	F
cDNA	c.1000G>A, c.643C>T	c.1000G>A, c.72dupT	c.78G>A, c.463G>A	c.60T>A, c.60T>A	c.59A>C, c.1000G>A	c.709A>T, c.803T>C	c.803T>C, c.803T>C	c.1000G>A, c.59A>C	c.709A>T, c.709A>T,	c.803T>C, c.928G>A	c.928G>A, c.928G>A	c.803T>C, c.803T>C	c.1006G>A, c.10006>A	c.889C>A, c.889C>A	c.1162C>T,c.803T>C	NA	NA
																		protein change	p.(Ala334Thr),p.(Arg215*)	p.(Ala334Thr),p.(Gly25Trpfs*55)	p.(Lys26Lys),p.(Ala155Thr)	p.(His20Gln),p.(His20Gln)	p.(His20Pro),p.(Ala334Thr)	p.(Thr237Ser),p.(Ile268Thr)	p.(Ile268Thr),p.(Ile268Thr)	p.(Ala334Thr),p.(His20Pro)	p.(Thr237Ser),p.(Thr237Ser)	p.(Ile268Thr),p.(Val310Met)	p.(Val310Met),p.(Val310Met)	p.(Ile268Thr),p.(Ile268Thr)	p.(Gly336Ser),p.(Ala334Thr)	p.(Leu297Ile),p.(Leu297Ile)	p.(Arg388*),p.(Ile268Thr*)	NA	NA
Clinical features																																			
Age at onset	fetus at 29 weeks	2 weeks	0.5 month	0 month	0 month	0 month	0 month	0 month	5 days	At birth	At birth	0 month	10 days	prenatal	At birth	At birth	
Age at diagnosis	NA	5 years	32 months	1 year	8 years	1 year	0 year	48 years	36 days	2 weeks	26 days	2 months	3.5months	At birth		1 year	3 weeks
Outcome	Died at the age of 4 months	Alive until 26 years	Alive until 8 years	Alive until 16 years	Alive until 43 years	Alive until 26 years	NA	Alive until 51 years	Alive until 6 months	Alive	Died at the age of 3 months	NA	Died at the age of 7.5 months	Alive until 34 months	Died at the age of 8 weeks	Alive until 10 years	NA
																		Periodic fever	+	+	s+	+	+	+	-	+	-	+	+	+	+	+	+	+	+
Skin rash	+	+	+	-	-	+	-	-	-	-	+	+	-	-	+	-	-
Lymphadenopath-y	+	+	+	-	-	+	-	-	-	-	+	+	+	-	-	-	-
Hepatosplenomeg-aly	+	+	+	-	+	+	+	-	-	+	+	+	+	+	+	+	+
Joint pain	+	+	+	+	-	-	-	-	-	-	-	-	+	-	-	-	-
Digestive tract symptoms (vomiting, diarrhea)	+	+	+	+	+	-	-	+	+	+	+	+	+	+	+	-	+
Neurological symptoms																	
Developmental retardation	+	+	-	+	+	+	-	+	-	+	NA	+	+	+	+	+	NA
Cerebellar ataxia	+	+	-	-	+	+	-	+	-	+	NA	-	+	-	-	-	NA
Hypotonia	+	+	-	+	-	-	-	-	+	+	-	+	-	-	-	-	NA
Growth and development																	
Hypogenesis	-	+	+	+	-	+	-	-	-	-	-	+	-	+	+	+	+
Short stature	-	-	+	+	-	+	-	-	+	+	-	+	+	-	-	+	+
Ocular symptoms																	
Retinal degeneration	+	-	-	-	+	+	-	+	-	-	-	+	+	-	-	-	-
Cataract	-	-	+	-	-	+	+	-	-	+	-	+	+	-	-	+	+
Others																	
Acute rhabdomyolysis	-	-	-	-	-	-	-	-	-	-	-	-	-	-	-	-	-
Myocardial hypertrophy	-	-	-	-	-	-	-	-	-	-	-	-	-	-	-	-	-
Imaging manifestations																	
Spinocerebellar ataxia	+	+	-	-	+	-	-	-	-	+	-	+	+	NA	NA	+	NA
Cerebellar agenesia hypoplasia	+	-	-	-	-	+	+	-	-	+	-	+	+	NA	NA	-	NA
Atrophy of the cerebral cortex	-	-	-	-	-	-	+	-	-	+	-	-	-	NA	NA	-	NA
Intracranial cystic lesions	-	+	-	-	-	-	+	-	+	-	-	-	-	NA	NA	-	NA
Reference	18264963	28 34145613	40717084	28 34145613	34145613	34145613	34145613	34145613	31096039	22405037	Novel	22271696	26965418	31651069	23146290	8352861	
(PMID)																	

P, patient; -, absent; +, observed; N/A, not available.

Corticosteroid therapy is effective in diminishing the severity of inflammatory attacks but fails to prevent disease crises. Potentially beneficial therapeutic options for the patient included interleukin (IL)-1 receptor antagonists (e.g., anakinra) or tumor necrosis factor-α (TNF-α) inhibitors (e.g., etanercept); however, access to these therapeutic agents was limited in our region. Furthermore, the patient’s parents declined the additional benefit of allogeneic bone marrow transplantation and requested discharge (6). After discharge, the patient suffered from recurrent diarrhea. Unfortunately, the patient’s family declined further medical evaluation and management, and the patient subsequently died at the age of 3 months due to dehydration and electrolyte disturbances.

## Discussion and conclusions

To date, 30 cases of MA have been reported worldwide, all of which range from neonates to 51 years of age, and only 16 neonates have been documented in the literature (5, 7–15). The genotypic and clinical phenotypic data of the neonates are summarized in Table 1.

Herein, we report a case of MA in one of the youngest neonates reported to date, characterized by early onset and diagnosis. This case was caused by a novel homozygous variant in the *MVK* gene, c.928G>A (p.Val310Met), which clinically mimicked neonatal infection. Moreover, the patient presented with normal body temperature after receiving prednisone acetate treatment. Repeatedly elevated inflammatory indicators, abnormal liver function, significant elevated mevalonic acid and mevalonolactone levels, recurrent fever, digestive symptoms, and poor weight gain were observed. No abnormalities were detected on the ophthalmological examination. Additionally, no central nervous system (CNS) involvement was observed in the patient prior to his death. The genotypic and clinical phenotypic spectrum of patients with MA is heterogeneous. Manifestations of the disease seem to be age dependent. A review of neonatal early-onset MA cases revealed that the majority of patients exhibited characteristic features including recurrent fever (15/17), hepatosplenomegaly (14/17), digestive symptoms (14/17), developmental retardation (12/17), neurological impairment (12/15), and abnormal cranial MRI findings (10/14). In approximately 50% of cases, patients presented with lymphadenopathy (7/17), skin rash (7/17), feeding difficulties (9/17), and ocular symptoms (11/17).

This case highlights that when neonates present with recurrent fever accompanied by vomiting, diarrhea, hepatosplenomegaly, and lymphadenopathy, clinicians should pay close attention to differentiating MA from infectious diseases to avoid misdiagnosis or underdiagnosis. A limitation of this case report is the lack of serum IL level assessment, which was attributable to parental preference against this investigative assay. Periodic fever and elevated inflammatory markers are associated with MVK enzyme activity (16). A previous study reported that minor elevations in temperature can set off a chain of events with MVK enzyme activity becoming progressively rate-limiting, leading to a temporary deficiency of isoprenoid end-products, which induces inflammation and fever (17). Lymphadenopathy may be related to the lymphocyte apoptosis defect (18). The potential underlying mechanism is that MA induces a predominance of type 2 helper T cells (Th2), leading to elevated levels of IL-4, IL-5, IL-6, TNF-α, and immunoglobulins (either hyper-IgD or hyper-IgE levels). Furthermore, the Th2 bias induced by MKD may be associated with cell signaling proteins and lipid rafts—assemblages of cholesterol and sphingolipids within the lipid bilayer. Most patients with MA exhibit elevated urinary leukotriene (LT) E4 excretion, which exhibits a positive linear correlation with increased mevalonic acid excretion. This elevation in urinary LTE4 level suggests enhancement of systemic cysteinyl leukotrienes synthesis.

Urinary mevalonic acid levels are used as a tool in the diagnostic process of MKD. The urine sample is collected during febrile episodes because the excretion of mevalonic acid is elevated during febrile episodes. Previous studies have reported that mildly increased mevalonic acid excretion is observed in patients of HIDS during febrile episodes, whereas severely affected patients with MA exhibit relatively low mevalonic acid excretion (4, 5). Therefore, the concurrent initiation of ELISA and WGS combined with Sanger sequencing should be performed for final diagnosis.

No curative drug treatment exists for MA, and individualized symptomatic treatments are primarily administered. Corticosteroid treatment is effective in diminishing the severity of attacks but cannot prevent disease crises. The patient may benefit from treatment with IL-1-receptor antagonists (e.g., anakinra) or TNF-α inhibitors (e.g., etanercept); however, access to these medicines was limited in our city. Hematopoietic stem cell transplantation is an effective treatment option for children who do not respond to drug therapy (6).

The case description further refines the clinical and molecular features of MA to provide a reference for genetic counseling and research on the diagnosis and treatment of this rare syndrome. Further observations and studies are expected to lead to a better understanding of the clinical manifestations related to *MVK* mutations and the development of translational knowledge that will impact the treatment and prognosis of afflicted patients. Early diagnosis of MA and initiation of effective treatment are crucial, and patients can choose different medications depending on the severity of the disease. This study provides new perspectives and therapeutic directions for targeted drug development and treatment of monogenic diseases.

## Data Availability

The raw data supporting the conclusions of this article will be made available by the authors, without undue reservation.

## References

[1] Esmeraldo MA Prates IR Lucato LT Barbosa Junior AA . Mevalonic aciduria in a pediatric patient: A case report and literature review of neuroimaging findings. Cureus. (2024) 16:e65209. doi: 10.7759/cureus.65209, PMID: 39176373 PMC11340854

[2] Jiang M Liu L Mei H Li X Cheng J Cai Y . Detection of inborn errors of metabolism using GC-MS: over 3 years of experience in southern China. J Pediatr Endocrinol Metab. (2015) 28:375–80. doi: 10.1515/jpem-2014-0164, PMID: 25781538

[3] Federici S Sormani MP Ozen S Lachmann HJ Amaryan G Woo P . Evidence-based provisional clinical classification criteria for autoinflammatory periodic fevers. Ann Rheum Dis. (2015) 74:799–805. doi: 10.1136/annrheumdis-2014-206580, PMID: 25637003

[4] Poll-The BT Frenkel J Houten SM Kuis W Duran M de Koning TJ . Mevalonic aciduria in 12 unrelated patients with hyperimmunoglobulinaemia D and periodic fever syndrome. J Inherit Metab Dis. (2000) 23:363–6. doi: 10.1023/a:1005635431364, PMID: 10896295

[5] Prasad C Salvadori MI Rupar CA . Severe phenotypic spectrum of mevalonate kinase deficiency with minimal mevalonic aciduria. Mol Genet Metab. (2012) 107:756–9. doi: 10.1016/j.ymgme.2012.10.019, PMID: 23146290

[6] Neven B Valayannopoulos V Quartier P Blanche S Prieur AM Debre M . Allogeneic bone marrow transplantation in mevalonic aciduria. N Engl J Med. (2007) 356:2700–3. doi: 10.1056/NEJMoa070715, PMID: 17596604

[7] Brennenstuhl H Nashawi M Schroter J Baronio F Beedgen L Gleich F . Phenotypic diversity, disease progression, and pathogenicity of MVK missense variants in mevalonic aciduria. J Inherit Metab Dis. (2021) 44:1272–87. doi: 10.1002/jimd.12412, PMID: 34145613

[8] Ruiz Gomez A Couce ML Garcia-Villoria J Torres A Bana Souto A Yague J . Clinical, genetic, and therapeutic diversity in 2 patients with severe mevalonate kinase deficiency. Pediatrics. (2012) 129:e535–539. doi: 10.1542/peds.2010-2192, PMID: 22271696

[9] Schwarzer V Haas D Hoffmann GF Meyberg H Gembruch U . Abnormal prenatal ultrasound findings in mevalonic aciduria. Prenat Diagn. (2008) 28:257–8. doi: 10.1002/pd.1917, PMID: 18264963

[10] Chaudhury S Hormaza L Mohammad S Lokar J Ekong U Alonso EM . Liver transplantation followed by allogeneic hematopoietic stem cell transplantation for atypical mevalonic aciduria. Am J Transplant. (2012) 12:1627–31. doi: 10.1111/j.1600-6143.2011.03989.x, PMID: 22405037

[11] Mancini J Philip N Chabrol B Divry P Rolland MO Pinsard N . Mevalonic aciduria in 3 siblings: a new recognizable metabolic encephalopathy. Pediatr Neurol. (1993) 9:243–6. doi: 10.1016/0887-8994(93)90095-t, PMID: 8352861

[12] Guan C Wang W Zhou Q Sun J Liu L Liu L . Mevalonate kinase deficiency: genetic and clinical characteristics of a Chinese pediatric cohort. Pediatr Rheumatol Online J. (2025) 23:78. doi: 10.1186/s12969-025-01131-1, PMID: 40717084 PMC12297814

[13] Pietrasanta C Minoia F Torreggiani S Ronchi A Gattorno M Volpi S . When neonatal inflammation does not mean infection: an early-onset mevalonate kinase deficiency with interstitial lung disease. Clin Immunol. (2019) 205:25–8. doi: 10.1016/j.clim.2019.05.002, PMID: 31096039

[14] Erdol S Cekic S Kilic SC Saglam H Kilic SS . Massive ascites in a canakinumab resistant case with MVA leading to bone marrow transplantation. Rheumatol Int. (2016) 36:1011–3. doi: 10.1007/s00296-016-3456-9, PMID: 26965418

[15] Szymanski AM Davila Saldana B Ferreira CR Loechelt B Jung L . Mevalonic aciduria: Does stem cell transplant fully cure disease? Pediatr Transplant. (2020) 24:e13604. doi: 10.1111/petr.13604, PMID: 31651069

[16] Boursier G Rittore C Milhavet F Cuisset L Touitou I . Mevalonate kinase-associated diseases: hunting for phenotype-genotype correlation. J Clin Med. (2021) 10:1552. doi: 10.3390/jcm10081552, PMID: 33917151 PMC8067830

[17] Houten SM Frenkel J Rijkers GT Wanders RJ Kuis W Waterham HR . Temperature dependence of mutant mevalonate kinase activity as a pathogenic factor in hyper-IgD and periodic fever syndrome. Hum Mol Genet. (2002) 11:3115–24. doi: 10.1093/hmg/11.25.3115, PMID: 12444096

[18] Bodar EJ van der Hilst JC van Heerde W van der Meer JW Drenth JP Simon A . Defective apoptosis of peripheral-blood lymphocytes in hyper-IgD and periodic fever syndrome. Blood. (2007) 109:2416–8. doi: 10.1182/blood-2005-10-039578, PMID: 17138829

