# Supplementary material for: Case Report: Early-onset mevalonic aciduria in neonates with inflammatory marker elevated
Source: Front Immunol. 2026 Mar 5;17:1773017. doi: 10.3389/fimmu.2026.1773017 (PMC12999955; doi:10.3389/fimmu.2026.1773017)

Fig S1 The serial measurements of CRP, hemoglobin, and platelet counts in the patient. Routine blood tests demonstrated the levels of CRP (A), hemoglobin (B) and platelet count (C) on different days of life (DOL).


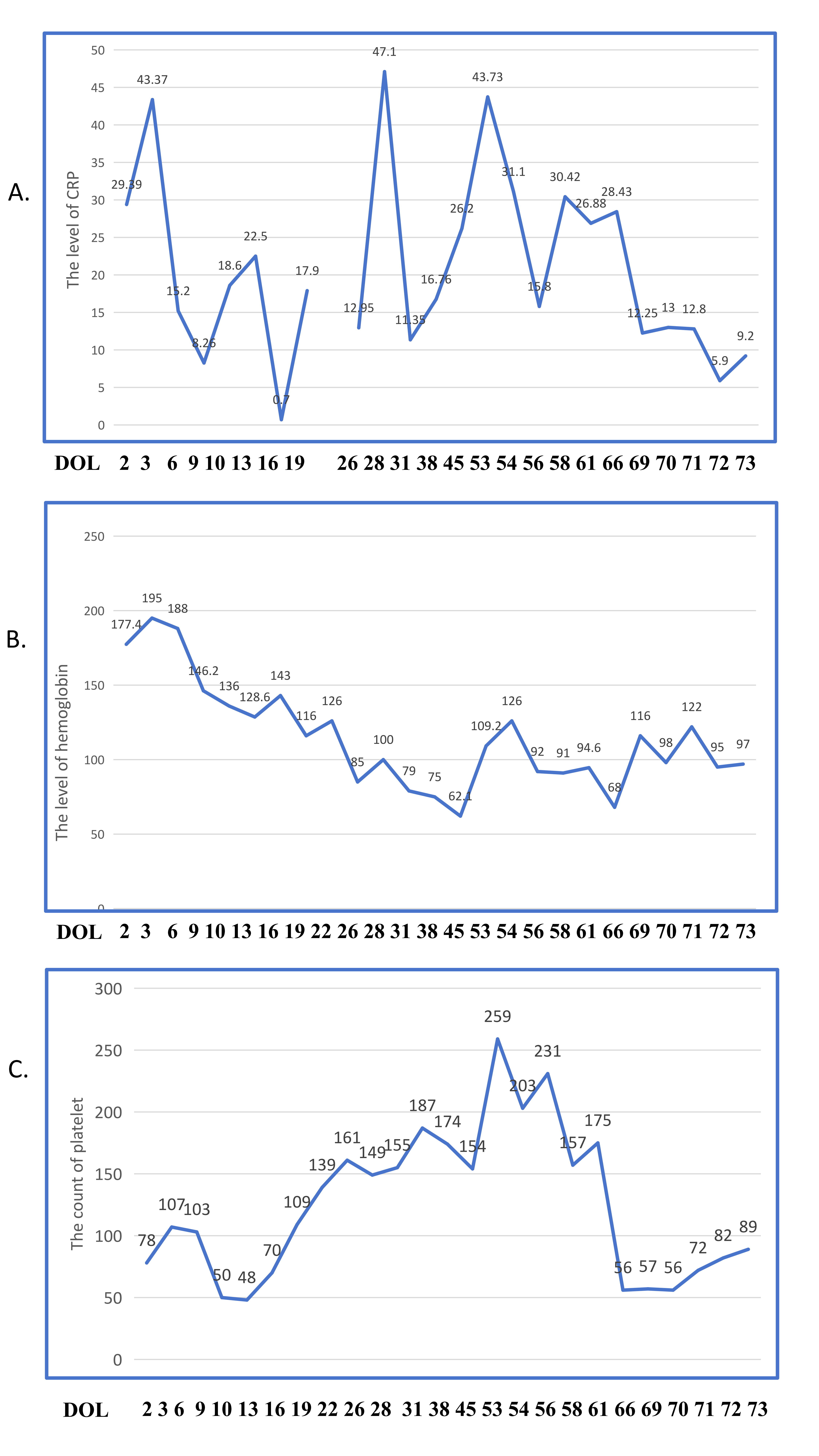


Fig S2 The genome sequencing of the patient and his parents. Genome sequencing demonstrated that there was a homozygous mutation (c.928G>A, p.V310M) in the *MVK* gene in the patient (A),and a heterozygous variation at this locus in his father (B) and mother (C).


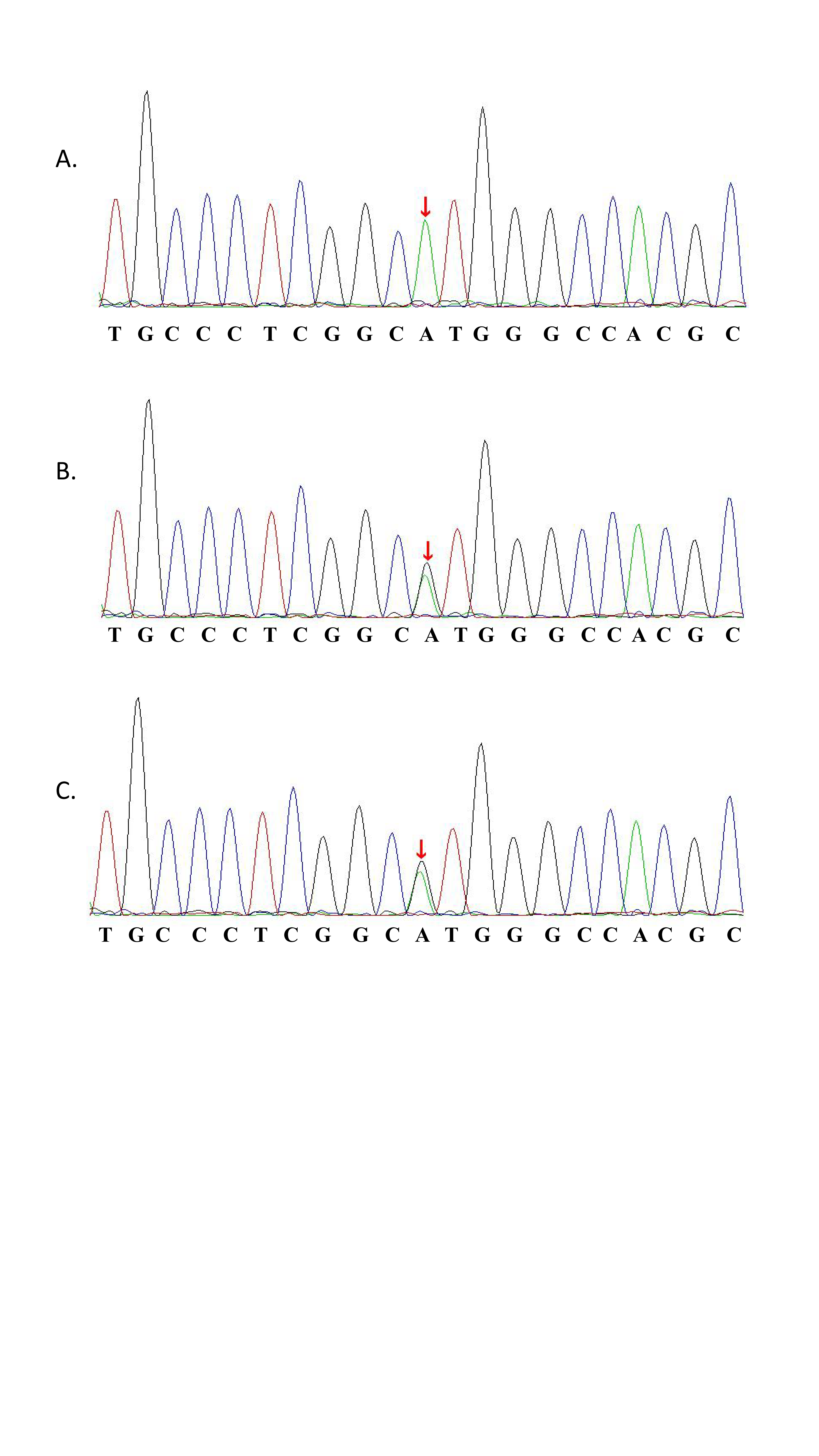

Supplement: Supplementary file 1 [file DataSheet1.docx]
